# Corn starch/Carboxymethyl Cellulose Films: Influence of Citric Acid on Water Susceptibility, Morphological and Tensile Properties

**DOI:** 10.1021/acsomega.5c08949

**Published:** 2026-01-21

**Authors:** Michele Barbosa Santos, José Antonio da Silva Souza, Verônica Scarpini Cândido, Jordan Del Nero, Waldomiro Paschoal Junior, Ana Aurea Barreto Maia, Bruno Marques Viegas, Severino Alves Junior, Carlos Alberto Brito da Silva, Marcos Vinícius da Silva Paula

**Affiliations:** † Faculdade de Engenharia de Materiais, Universidade Federal do Pará, Ananindeua 67130-660, Brasil; ‡ Programa de Pós-Graduação em Engenharia de Recursos Naturais da Amazônia, Universidade Federal do Pará, Belém 66079-420, Brasil; § Programa de Pós-Graduação em Ciência e Engenharia de Materiais, Universidade Federal do Pará, Ananindeua 67130-660, Brasil; ∥ Faculdade de Física, Universidade Federal do Pará, Belém 66075-110, Brasil; ⊥ Faculdade de Engenharia Química, Universidade Federal do Pará, Belém 66075-110, Brasil; # Programa de Pós-Graduação em Biotecnologia, Universidade Federal do Pará, Belém 66075-110, Brasil; ∇ Departamento de Química Fundamental, Universidade Federal de Pernambuco, Recife 50740-560, Brasil; ○ Faculdade de Física, Universidade Federal do Pará, Ananindeua 67130-660, Brasil

## Abstract

The present study aimed to produce and characterize corn starch/carboxymethyl cellulose (TPC/CMC) films cross-linked with different concentrations of citric acid (CA). The films were obtained by solvent evaporation (casting solution) with 5, 10, 15, and 20% citric acid relative to the CMC mass (TPC/CMC-CA-0 to 20). They were qualitatively evaluated for visual aspects, swelling, moisture content, and solubility, and characterized by Fourier transform infrared spectroscopy (FTIR), thickness, and scanning electron microscopy (SEM). The results allowed us to evaluate the cross-linking effect promoted by citric acid. Visually, all films showed excellent optical transparency. FTIR spectroscopy demonstrated the formation of ester groups, indicative of cross-linking of the TPC-CMC films. SEM images revealed a homogeneous surface morphology without porosity in the films with CA. The addition of CA to the TPC-CMC films reduced water swelling, moisture absorption, and permeation. Additionally, the insertion of citric acid promoted an improvement in the tensile strength of TPC-CMC from 6.07 to 8.39 MPa for TPC-CMC/CA-15. This same trend was obtained with an increase of around 115% for the modulus in the TPC-CMC/CA-15 film compared to the film without CA. These results were due to cross-linking promoted by CA which produces a more cohesive material and thus increases the modulus and tensile strength. Thus, our results demonstrate that the cross-linking promoted by CA in TPC-CMC films has a profound influence on their water susceptibility and tensile properties.

## Graphical abstract

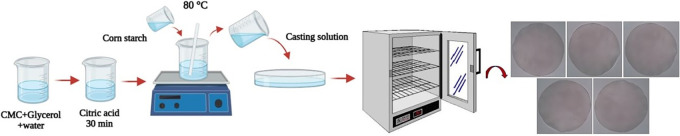

## 1 Introduction

In the context of sustainability and solid waste management, biodegradable polymers have emerged as a focal point of scientific and technological research.^1,2^ These materials are principally characterized by their ability to undergo decomposition via the metabolic processes by microorganisms as bacteria, fungi, and algae.^3^ This intrinsic degradability differentiates biodegradable polymers from conventional petroleum-derived materials such as polyethylene and polypropylene, which persist in the environment and contribute substantially to contemporary ecological challenges (Hernández et al., 2022).^4^ Classification of biodegradable polymers is typically based on their origin, delineating them as either natural or synthetic. Natural polymers, or biopolymers, are biosynthesized by living organisms and derived from renewable sources, including starch, cellulose, and chitin.^5,6^

Among natural polymers, starch warrants particular attention. Starch functions as a primary energy reserve polymer in plants such as corn, potato, rice, and cassava, and is considered highly promising for the development of biodegradable polymers.^7,8^ The scientific interest in starch arises from its notable advantages, including renewability, low cost, complete biodegradability, and widespread global availability, particularly in agriculturally intensive regions such as Brazil. Starch is a high-molecular-weight, semicrystalline natural polymer composed of repeating glucose units linked by glycosidic bonds. Its structure and physicochemical properties are governed by the relative abundance of two glucose-based macromolecules: amylose and amylopectin.^9^

One of its most important properties is its gelation capacity, which involves the irreversible transformation of starch granules into a viscoelastic paste when subjected to heating and shearing in the presence of a plasticizer, such as glycerol.^10^ During gelation, the starch granule is destroyed, resulting in the formation of a continuous matrix, an essential characteristic for film production, which transforms starch into a material known as thermoplastic starch.^11^ However, these thermoplastic materials typically exhibit poor mechanical properties. One way to improve these properties is by adding biodegradable polymers, such as carboxymethyl cellulose (CMC), to starch.^12^

CMC is a polysaccharide derived from cellulose. CMC is a cellulose ether formed by the reaction of cellulose with chloroacetic acid in an alkaline medium. This reaction introduces carboxymethyl groups into some of the glucose units of cellulose, imparting an anionic nature and water solubility to the polymer.^13^ CMC is an ideal component for formulating films using thermoplastic starch. These two polymers are chemically compatible, favoring the formation of a more homogeneous mixture. CMC contains carboxyl groups that form strong hydrogen bonds with the hydroxyl groups of starch, forming an interpenetrated polymer network that is denser and more cohesive, resulting in a significant improvement in the material properties.^14^ However, CMC-derived materials generally exhibit high water solubility. One solution to this problem is to cross-link CMC with cross-linking agents, forming cross-links between the polymer chains.^15^ The formation of cross-links reduces the water solubility and improves the mechanical and barrier properties.^15^ Citric acid is a prominent cross-linking agent for biopolymers. This acid is nontoxic, biodegradable, and renewable. This organic tricarboxylic acid has the characteristic of forming cross-links with polysaccharides through esterification between its carboxyl groups and the hydroxyl groups of the biopolymers.^16^ Several efforts have been made to reduce the water solubility of natural polymers through cross-linking. Hydroxyethyl tamarind gum-based hydrogel films with improved mechanical properties and swelling were developed by esterification with citric acid.^17^ Citric acid was used as a cross-linking agent to optimize the thermal properties, vapor permeation, and swelling of carboxymethyl chitosan/poly­(vinyl alcohol)” films.^18^ CMC and poly­(vinyl alcohol)” films were cross-linked with citric acid and cupric chloride, and the cross-linking promoted a considerable improvement in the mechanical properties.^19^ Ghanbarzadeh et al., reported the preparation of TPC/CMC films with citric acid.^20^ However, they did not access the morphological properties of the films and the interactions established between their components.

Thus, our first objective in this study was to obtain corn starch films with cross-linked CMC with different citric acid contents, as a cross-linking agent, via the solvent casting method. Additionally, our main objective was to assess the influence of the addition of different citric acid contents to the polymer matrix on the tensile and morphological properties, as well as the water susceptibility of TPC-CMC films.

## 2 Experiment

### 2.1 Materials

All reagents were used without prior purification. Table 1 presents the description of the reagents used in this study.

**Table 1:** Description of the Reagents Used to Obtain the Films

reagent	description	supplier
corn starch	commercial cornstarch	Maizena (São Paulo, Brazil)
carboxymethyl cellulose sodium salt	carboxymethyl cellulose reagent grade 99.5%, viscosity 4000 cP −5000 cP, degree of substitution = 0.25^a^	ACS científica (São Paulo, Brazil)
glycerol	P.A. ACS 99.5%	Êxodo Científica (São Paulo, Brazil)
citric acid	anhydrous citric acid, P.A. 99.5%	Neon (São Paulo, Brazil)

a The degree of substitution was determined according to the methodology described in the literature.^21^

### 2.2 Methodology

#### 2.2.1 Films Preparation

Corn starch (TPC) films with CMC and different amounts of citric acid (5, 10, 15, and 20%), relative to CMC mass, were prepared using the solvent casting method. Initially, 1 g of CMC was dissolved in 50 mL of water using mechanical stirring for 30 min. Then, the previously determined amount of citric acid was added, followed by mechanical stirring until the mixture was homogenized. At the same time, 1 g of corn starch and 0.6 g of glycerol were mixed in another 50 mL of water with mechanical stirring for 30 min. Afterward, the starch solution was combined with the CMC solution, and the mixture was stirred for an additional 30 min at 80 °C to allow gelatinization. The solutions were transferred into Petri dishes (140 mm × 15 mm) and dried at 45 °C for 24 h. Once the drying process was complete, the films were carefully removed from the dishes and stored in a desiccator with silica for future characterization. Figure 1 provides a schematic representation of the steps used to obtain the films. Additionally, Table 2 describes the codes and composition of these films.

**Figure 1:**
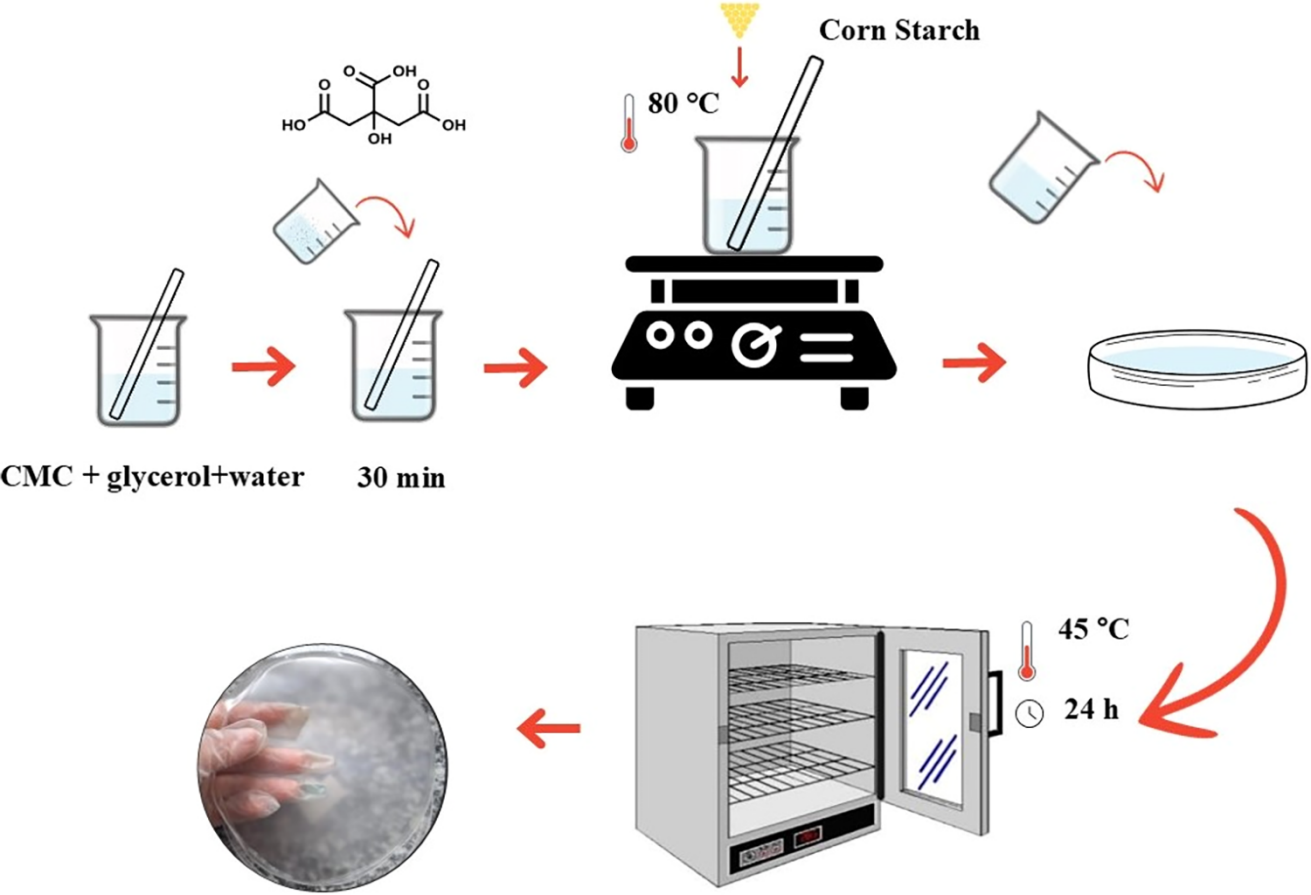
Schematic representation of the methodology used to obtain the films. First, dissolving the CMC and citric acid in water. Then, adding the CMC and citric acid solution to the starch until gelation at 80 °C. Adding the film-forming solution to the Petri dishes and then drying the films.

**Table 2:** Code and Composition for the Films

**code**	**composition**
TPC-CMC/CA-0	1 g of corn starch, 1 g of CMC, 0.6 g of glycerol and 100 mL of water
TPC-CMC/CA-5	1 g of corn starch, 1 g of CMC, 0.6 g of glycerol, 0.05 g of citric acid, and 100 mL of water
TPC-CMC/CA-10	1 g of corn starch, 1 g of CMC, 0.6 g of glycerol, 0.1 g of citric acid, and 100 mL of water
TPC-CMC/CA-15	1 g of corn starch, 1 g of CMC, 0.6 g of glycerol, 0.15 g of citric acid, and 100 mL of water
TPC-CMC/CA-20	1 g of corn starch, 1 g of CMC, 0.6 g of glycerol, 0.2 g of citric acid, and 100 mL of water

#### 2.2.2 Fourier Transform Infrared Spectroscopy (FTIR)

The vibrational modes of the films were accessed by acquiring spectra in the infrared region using a BRUKER model VERTEX 70v spectrophotometer. The spectra were collected via direct insertion of films using attenuated total reflectance (ATR), in the range of 4000 to 400 cm^–1^, utilizing 100 scans and a resolution of 1 cm^–1^.

#### 2.2.3 Scanning Electron Microscopy (SEM)

To analyze the surface morphology of the films, scanning electron microscopy (SEM) images were acquired using a TESCAN Mira3 electron microscope operating at 5 kV. For image acquisition, film samples were mounted on a stub holder and coated with a thin layer of gold using an Emitech model K675X Sputter Coater.

#### 2.2.4 Moisture Content (MC %)

To determine the moisture content (MC %), 2 cm × 2 cm samples were used. First, the initial weight of each sample (*m*_*i*_) was recorded. The samples were then dried at 105 °C for 24 h, after which they were weighed again *(m*_*f*_). The moisture content was calculated using the eq 1. Three replicates of each sample were used.

1 $$MC\;\left(\%\right)=\left(\frac{m_{i}-m_{f}}{m_{i}}\right)\times 100$$

#### 2.2.5 Solubility (*S* %)

To calculate the solubility content (*S* %), samples measuring 1.5 × 4 cm were used. The films were initially dried at 100 °C for 24 h and then weighed (*m*_*i*_). After dehydration, the samples were immersed in 50 mL of distilled water at room temperature for 24 h. Following this immersion, the samples were dried again at 105 °C for 24 h and weighed once more (*m*_*f*_). The *S* % results were determined using eq 2. Three replicates were conducted to calculate the *S* %.^34^

2 $$S\;\left(\%\right)=\left(\frac{m_{i}-m_{f}}{m_{i}}\right)\times 100$$

#### 2.2.6 Transparency

The transparency (*T*_r_) of the samples was performed in a UV–vis spectrophotometer by acquiring the percentage of transmittance (*T* %) at 600 nm and determined by eq 3.^34^ Three replicates were conducted to each sample.

3 $$T_{r}=\frac{\log\left(T\%\right)}{y}$$

Where, *y* is the thickness of the sample.

#### 2.2.7 Swelling in Water

Films measuring 2 cm × 2 cm were used to calculate swelling in water (SW %). Initially, the films were weighed (*m*_*i*_) and then submerged in 100 mL of water for 1, 2, 3, 4, 5, and 24 h. After each time interval, the films were removed from the water, gently dried with paper, and weighed again (*m*_*f*_). Three replicates were performed for each sample, and the SW % was calculated using eq 4.

4 $$\mathrm{SW}\;\left(\%\right)=\left(\frac{m_{f}-m_{i}}{m_{i}}\right)\times 100$$

#### 2.2.8 Water Vapor Permeability

The E96–95 method (American Society for Testing and Materials) was used to determine water vapor permeation.^22^ For each film, a cup containing anhydrous calcium chloride was coated with the film, maintaining a distance of 1–1.5 cm between the anhydrous solid and the film. After that, the cups were stored in a desiccator with a saturated sodium chloride solution. The mass of the cup-film system was monitored every 24 h until 216 h. The experiment was performed in a gradient of 75% relative humidity (RH) at 25 °C. Three replicates were performed for each sample and the water vapor permeability was determined using eq 5.

5 $$WVP=\left[\frac{t}{P_{st}\left({RH}_{des}-{RH}_{cup}\right)}\right]\left(\frac{\Delta_{m}}{\Delta_{t}\cdot area}\right)$$

WVP is the water vapor permeability, *t* is the thickness of the film, *P*_st_ is the saturation pressure for the water, RH_des_ is the relative humidity in the desiccator, RH_cup_ is the relative humidity in the cup, Δ*m*/Δ*t* is the slope of the mass curve, and area is the permeation area of the film.

#### 2.2.9 Tensile Properties

Tensile properties of TPA and NC films were evaluated in an INTERMERIC iM50, city of Mogi das Cruzes-SP, Brazil, universal mechanical testing machine with a 5 kN load cell and tensile speed of 5 mm/min, and an initial distance between jaws of 50 mm. The acquisition of the tensile properties of TPC/CMC films was carried out based on the ASTM D882 standard, with modifications. The films were cut into strips with a width of 25.0 mm and a length of 75.0 mm. The evaluated properties were: tensile strength (σ), the modulus of elasticity (*E*), and elongation at maximum strength (ε). Five replicates were performed for each film type.^23^

#### 2.2.10 Statistical Analysis

In this study, Duncan’s test was employed to evaluate significant statistical diferences in the data obtained from the films, with a significance level of 5% (*p* < 0.05).

## 3 Results and Discussion

### 3.1 Fourier Transform Infrared (FTIR) Spectroscopy

The characteristic vibrational modes of the functional groups present on the surface of the films were evaluated using FTIR. Figure 2 presents the FTIR spectra of carboxymethyl cellulose (CMC), arrowroot starch, and citric acid powders. For CMC, distinct vibrational modes were identified. These included a band at 3300 cm^–1^ corresponding to O–H stretching, bands at 2947–2855 cm^–1^ associated with C–H bonds, and bands at 1597 cm^–1^ and 1443 cm^–1^ ascribed to the asymmetric and symmetric stretching of carboxylate (COO^–^) groups.^24^ In arrowroot starch, the spectrum exhibited characteristic O–H stretching at 3315 cm^–1^ and C–H stretching at 2939 cm^–1^. The band at 1018 cm^–1^ was assigned to C–O stretching.^25^ The FTIR spectrum of citric acid displayed characteristic absorption bands at 3314 cm^–1^, related to O–H stretching. The band at 1709 cm^–1^ corresponded to C=O stretching vibrations.^26^

**Figure 2:**
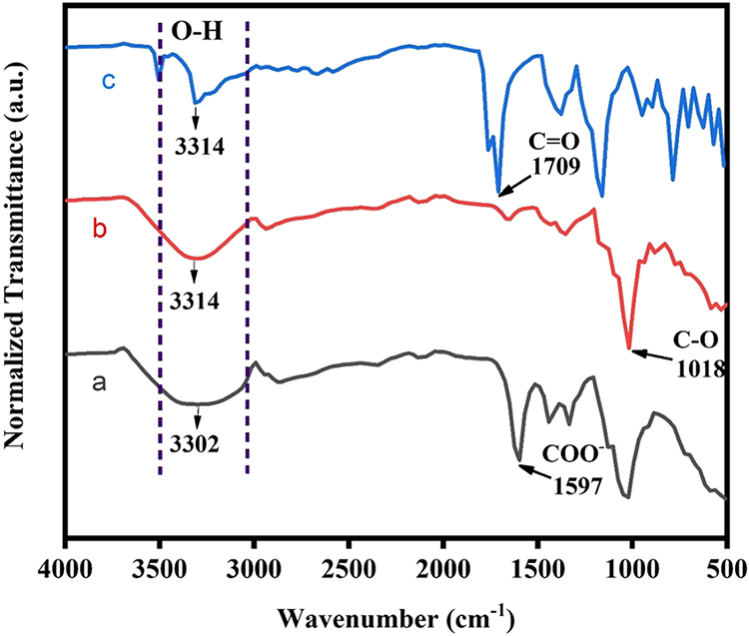
FTIR spectra for (a) CMC, (b) arrowroot starch, and (c) citric acid.

Figure 3 displays the FTIR spectra for TPC-CMC/CA-0, TPC-CMC/CA-5, TPC-CMC/CA-10, TPC-CMC/CA-15, and TPC-CMC/CA-20. In general, the spectra for the TPC-CMC films with citric acid exhibited similar spectral behavior with absorption bands characteristic of starch and CMC. For the band centered at 3298 cm^–1^, the presence of OH groups derived from starch, CMC, glycerol, and also moisture was observed.^12^ C–H bonds were observed in the bands around 2947 and 2890 cm^–1^.^27^ The bands observed in the region of 1730 to 1750 cm^–1^ were described as stretching vibrations of the carbonyl group characteristic of ester and citric acid.^15^ This band is not observed for TPC-CMC/CA-0, which is indicative of the presence of citric acid in the other samples. These results indicate that ester bonds have formed between OH groups of starch and CMC with carboxyl groups of citric acid, as previously reported for superabsorbent cross-linked carboxymethyl cellulose-PEG hydrogels.^24^ Similar results were reported for starch that has been cross-linked with citric acid. Additionally, the bands in the regions of 1620–1597 and 1435–1405 cm^–1^ are derived from the asymmetric and symmetric stretching of the COO^–^ group, respectively.^9^ Furthermore, the bands in the region of 1620–1610 cm^–1^ can also overlap with bending vibrations of OH groups due to residual moisture.^9^ The bands observed Around 1040 and 930 cm^–1^ are attributed to the stretching of the C–O bond. Our results are in good agreement with those observed for corn starch and cassava films with CMC.^14^ However, the authors in their study did not assess the impact of adding citric acid to these films.

**Figure 3:**
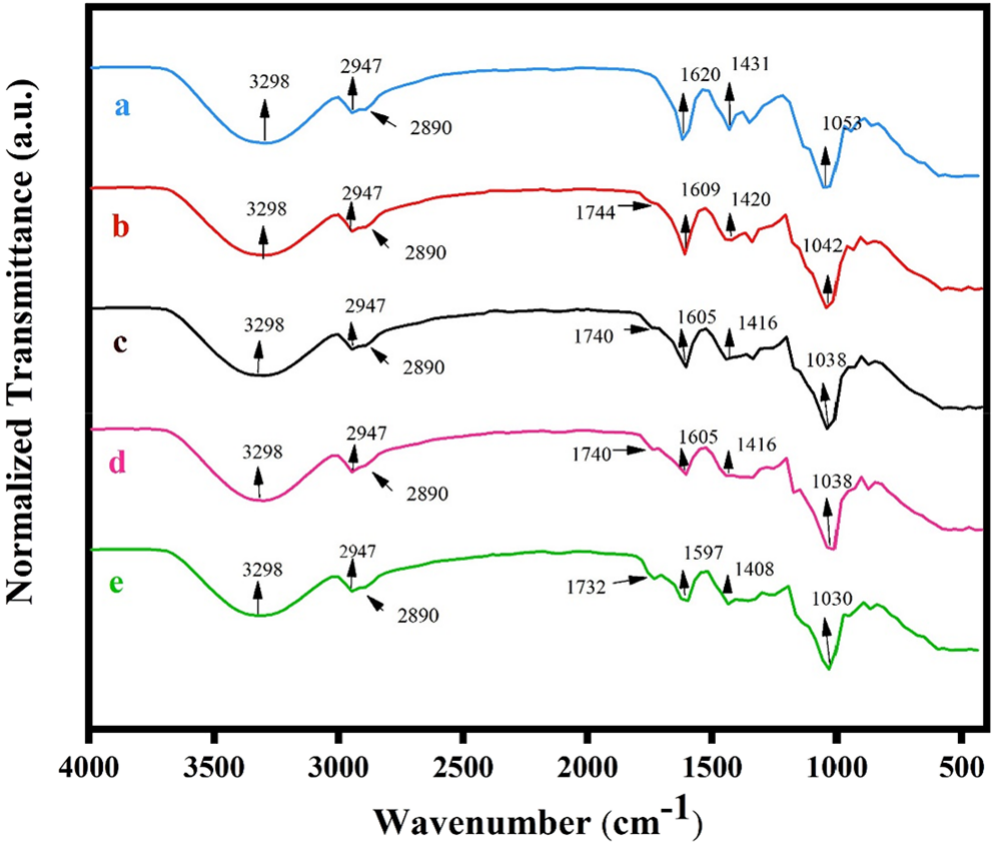
FTIR spectra for (a) TPC-CMC/CA-0; (b) TPC-CMC/CA-5; (c) TPC-CMC/CA-10, (d) TPC-CMC/CA-15, and (e) TPC-CMC/CA-20.

### 3.2 Scanning Electron Microscopy (SEM)

SEM images were acquired to assess the surface morphology of TPC-CMC films with and without citric acid, as shown in Figure 4. In general, the films with and without citric acid displayed a smooth surface, free of pores, bubbles, fractures, or any signs of immiscibility between their componentes, as observed in the visual appearance results. Nevertheless, a few characteristic nonplasticized starch granules were observed on the surface of the films (Figure 4a).^28^ The results can be attributed to the good chemical compatibility between the film polymers, as both are formed by intra- and intermolecular hydrogen bonds that promote this compatibility. Additionally, the inclusion of citric acid did not alter the surface morphology of the films. However, at higher magnifications, dispersed granules can be seen on the film surface, which are attributed to nonsolubilized starch and CMC. In Figure 4g, the dark region separating the dark area from the rest of the matrix attributed to a region of lower compatibility between starch and CMC.^29^

**Figure 4:**
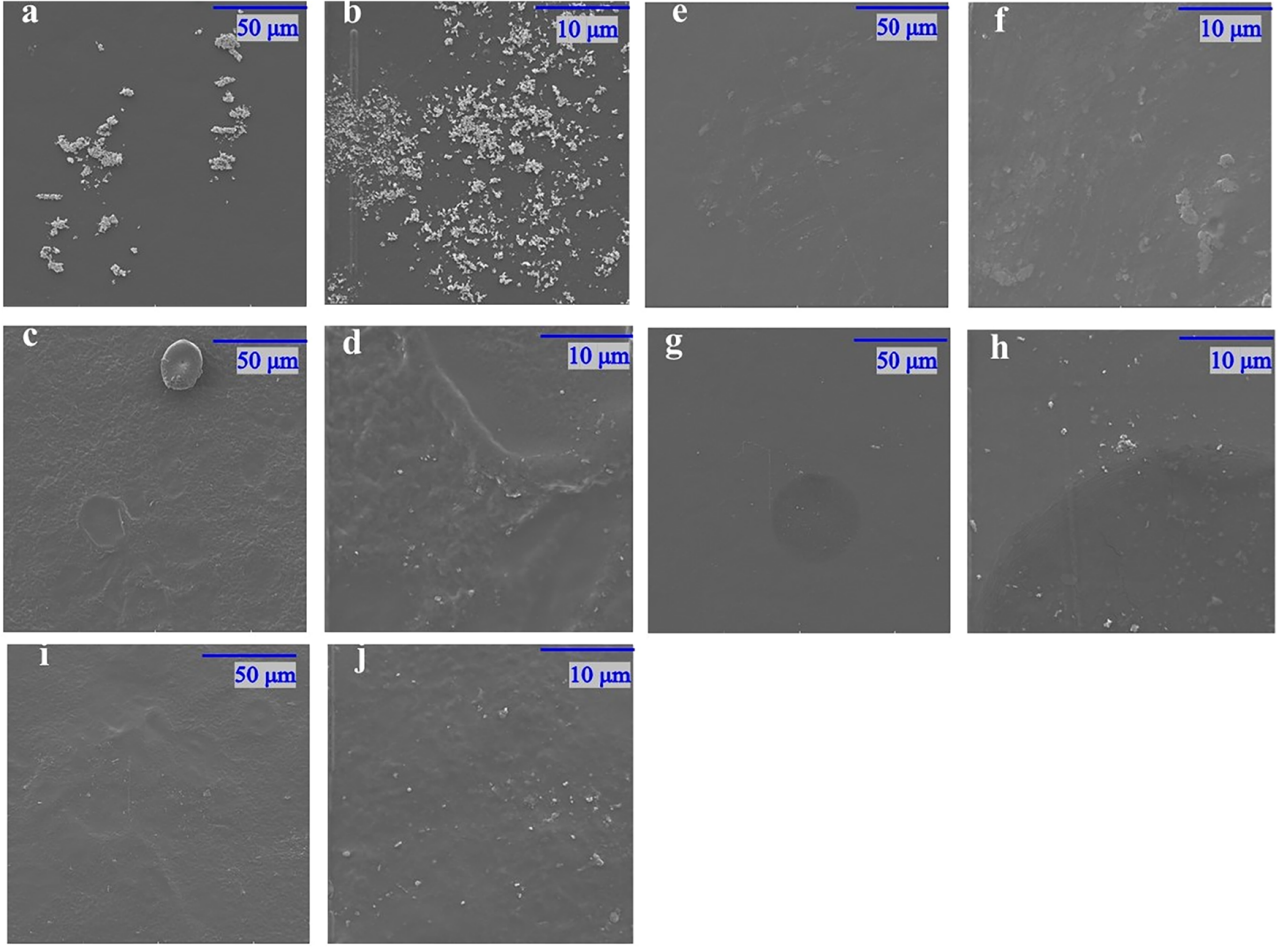
SEM images for (a, b) TPC-CMC/CA-0, (c, d) TPC-CMC/CA-5, (e, f) TPC-CMC/CA-10, (g, h) TPC-CMC/CA-15, and (i, j) TPC-CMC/CA-20.

Additionally, the presence of fewer grains in TPC-CMC films with citric acid can be attributed to its plasticizing effect, as previously reported by Ghanbarzadeh et al.^20^ These results are consistente with those reported for corn and cassava starch with CMC films.^14^

### 3.3 Moisture Content, Water Solubility, Water Vapor Permeability, and Swelling in Water

The moisture content provides the percentage of water mass present in the films.^30^ Figure 5a presents the MC (%) results forTPC-CMC/CA-0, TPC-CMC/CA-5, TPC-CMC/CA-10, TPC-CMC/CA-15, and TPC-CMC/CA-20. In our investigation to determine MC %, three replicates were used for each film type. Figure 5a shows that the TPC-CMC film without citric acid had the highest moisture content value (16%). The data in Figure 5a indicate that adding citric acid to the TPC-CMC films led to a reduction in MC %. These results can be attributed to the cross-linking reaction of the OH groups in the films with citric acid, as explained in the FTIR section, Cross-linking reactions involving citric acid result in a denser polymer matrix, which reduces water absorption in films,^31^ as previously observed for stale bread particle films treated with citric acid.^32^ These results demonstrate that the addition of citric acid to TPC-CMC films promotes a decrease in MC % due to its action as a cross-linking agent between the polymer chains.

**Figure 5:**
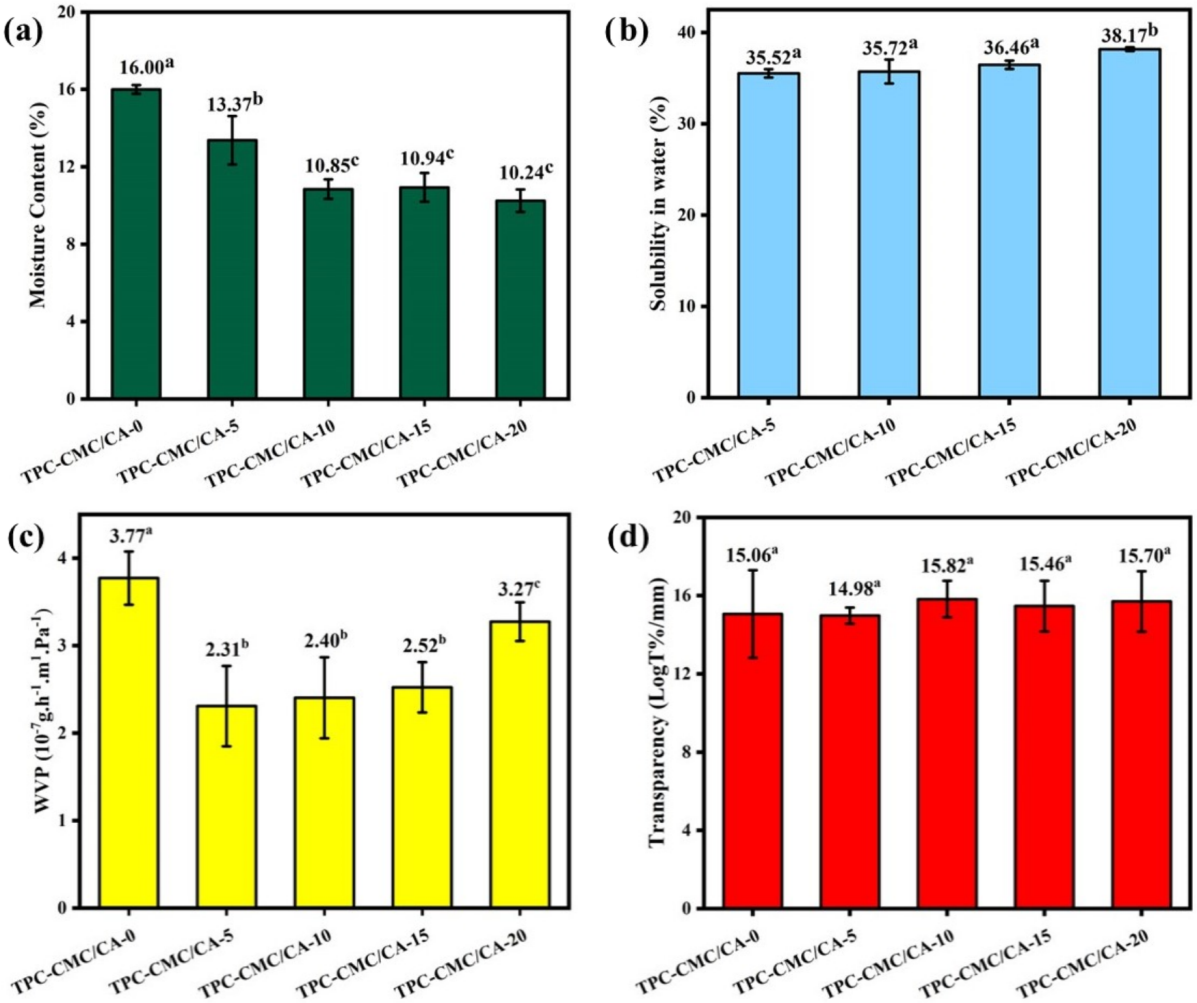
(a) moisture content; (b) solubility in water; (c) water vapor permeability; and (d) transparency for TPC-CMC/CA-0, TPC-CMC/CA-5, TPC-CMC/CA-10, TPC-CMC/CA-15, and TPC-CMC/CA-20. Means followed by equal letters did not differ (*p* < 0.05) by Duncan’s test.

The solubility of films in water is a crucial characteristic, as it assesses their resistance to disintegration upon contact with water.^33^ Figure 5b displays the results of *S* % for the films. For the TPC-CMC film without citric acid, we were unable to determine the *S* (%) value due to the film disintegrating during the test. This is attributed to the high hydrophilicity of the films, which facilitates the diffusion of water between the polymer chains and thus causes their dissolution.^34^ This finding can be explained by the lack of cross-linking reactions caused by the addition of citric acid.^34^ In contrast, for the samples containing citric acid, the test was completed successfully. It was observed that the TPC-CMC/CA-5 film had an *S* value of 35.53%, which was statistically similar to the TPC-CMC/CA-10, and TPC-CMC/CA-15 values. Furthermore, a slight increase was observed for TPA-CMC-20, with an *S* value of 37.94%. These results are in good agreement with previous studies reported for CMC films crossinked with citric acid for preserving freshness in bananas.^15^ Thus, our findings indicate that citric acid promoted the formation of cross-links between the polymer chains, which in turn hindered the disintegration of the films when exposed to water.

Hydrophilic polymers generally have high water vapor permeability, which is not suitable for food storage.^18^ The addition of citric acid influenced the WVP in TPC/CMC films, as shown in Figure 5c. The results in Figure 5c indicate that the TPC/CMC film exhibits the highest WVP value. For example, the addition of citric acid resulted in a 32% reduction in WVP for TPC-CMC/CA-10. These results indicate that the addition of citric acid promoted a decrease in WVP for TPC/CMC films. This behavior can be attributed to the formation of ester bonds between citric acid and OH groups of the matrix, as reported by Tavares et al.^12^ These bonds create a more organized structure in the biopolymer matrix, which in turn reduces the permeability of the films. Similar results were reported for guar gum/CMC films cross-linked with citric acid.^35^

TPC-CMC films with different citric acid contents were evaluated for their swelling in water for 1, 2, 3, 4, 5, and 24 h, as described in Figure 6. For each type of film, 3 replicates were performed using samples measuring 2 cm × 2 cm. For the TPC-CMC/CA-0 sample, this analysis could not be performed due to the rapid dissolution of the films upon immersion. The lack of polymeric cross-linking facilitated the penetration of water molecules into the matrix, leading to the dissociation of starch and CMC chains and subsequent dissolution.^15^ This observation aligns with prior studies, which have established that non-cross-linked starch films exhibit limited structural integrity in aqueous environments.^36^ Following 1 h of immersion in water, the TPC-CMC/CA-5 and TPC-CMC/CA-10 films displayed the highest swelling values (97.52% and 96.98%, respectively) (Figure 6). Conversely, the TPC-CMC/CA-10, TPC-CMC/CA-15, and TPC-CMC/CA-20 films exhibited a statistically significant linear decrease in swelling after 1 h of water exposure (Figure 6), suggesting that increased citric acid content is associated with reduced swelling. This trend demonstrates that citric acid serves as an efficient cross-linking agent, due to the enhanced density of ester bonds formed between polymer matrix chains.^35^ These new ester bonds hinder the penetration of water molecules, thus reducing film swelling.^35^ The same swelling profile was observed at 2, 3, 4, 5, and 24 h of contact with water. From a kinetic point of view, all films reached a swelling level close to equilibrium within 1 h of exposure, with few significant changes in subsequent times. This behavior is indicative of rapid diffusion of water into the polymer matrix.^37^ Thus, as observed by our results, the citric acid content significantly affects the swelling behavior of the TPC-CMC films.

**Figure 6:**
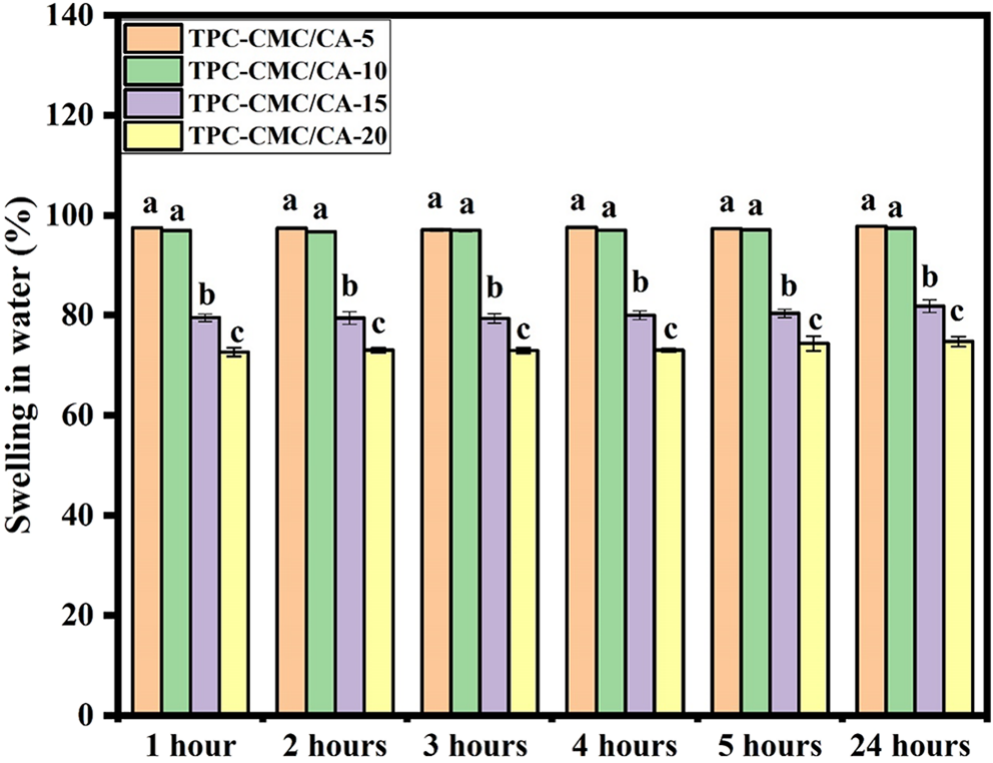
Swelling in water for TPC-CMC/CA-0, TPC-CMC/CA-5, TPC-CMC/CA-10, TPC-CMC/CA-15, and TPC-CMC/CA-20 at 1, 2, 3, 4, 5, and 24 h. Means followed by equal letters did not differ (*p* < 0.05) by Duncan’s test.

### 3.4 Transparency

TPC-CMC films with different amounts of citric acid were successfully prepared by the solvent cation method. These films were easily removed from the Petri dishes. As shown in Figure 7, the films exhibited a smooth surface without any bubbles or cracks, with an approximate thickness of 0.10 mm. Furthermore, all films demonstrated good homogeneity, which can be attributed to the strong chemical compatibility among the polymers.

**Figure 7:**
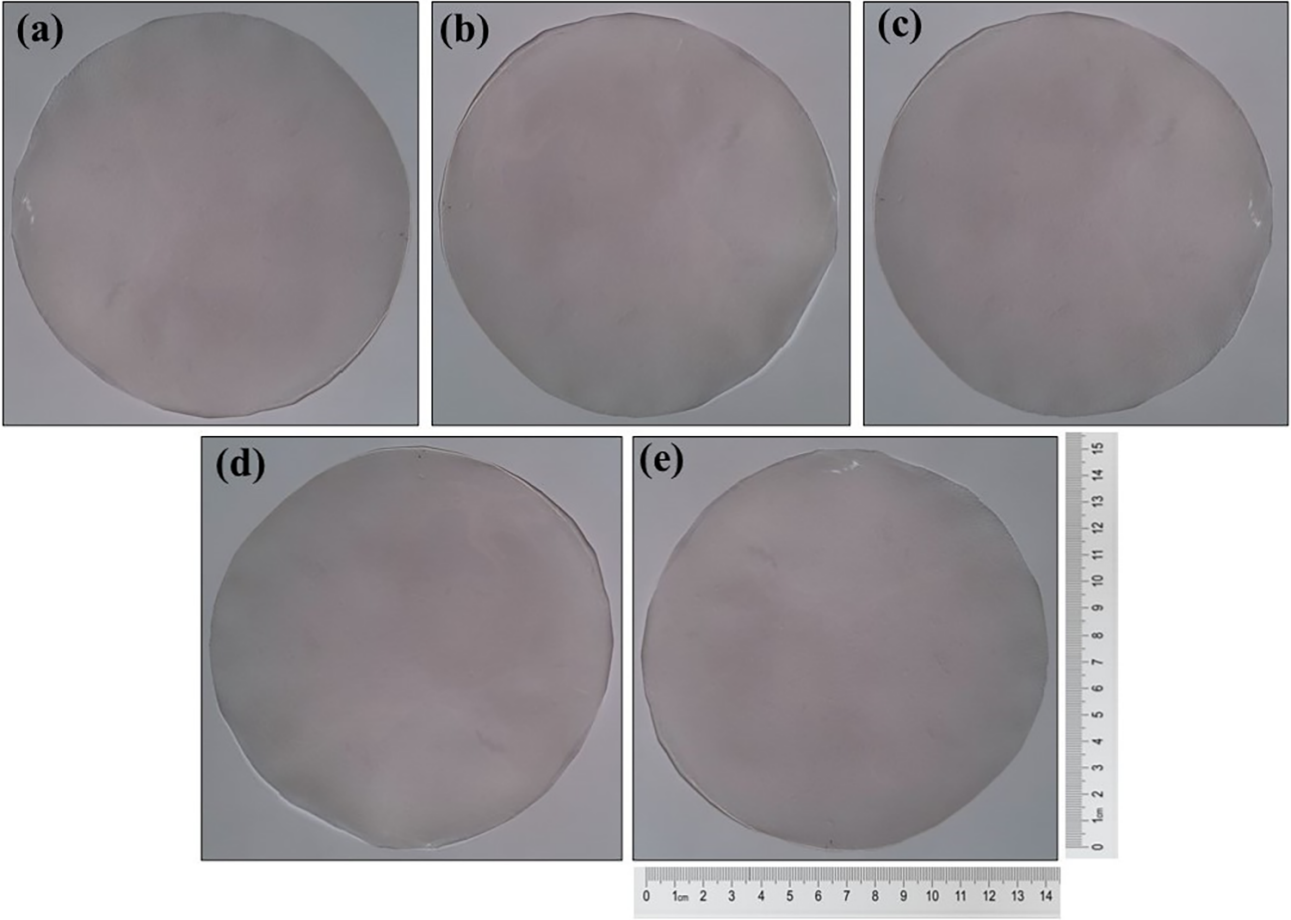
Visual aspect for (a) TPC-CMC/CA-0; (b) TPC-CMC/CA-5; (c) TPC-CMC/CA-10, (d) TPC-CMC/CA-15, and (e) TPC-CMC/CA-20.

Figure 5d depicts the average values for the transparency results. Based on Figure 5d, there is no statistically significant difference between the *T*_r_ values for the films analyzed. These results can be attributed to the chemical similarity between cornstarch and CMC, which promotes good miscibility and favors the formation of films with good transparency.^38^ Additionally, the inclusion of citric acid as a cross-linking agent did not affect the transparency of the films. These characteristics are ideal for food packaging applications because they enable visualization of the condition of stored food.^39^ These findings are in good agreement with those obtained for carboxymethyl cellulose- poly­(ethylene glycol) cross-linked with citric acid.^40^

### 3.6 Tensile Stress Test

Tensile properties are crucial for biopolymer films used in food packaging, as adequate strength and flexibility are required.^27^ Figure 8 shows the influence of citric acid cross-linking on the tensile properties of TPA-CMC films. The tested properties included tensile strength (σ), elongation at maximum strength (ε), and modulus of elasticity (*E*). Significant statistical variations were evaluated using the Duncan test, and three replicates were performed for each sample.

**Figure 8:**
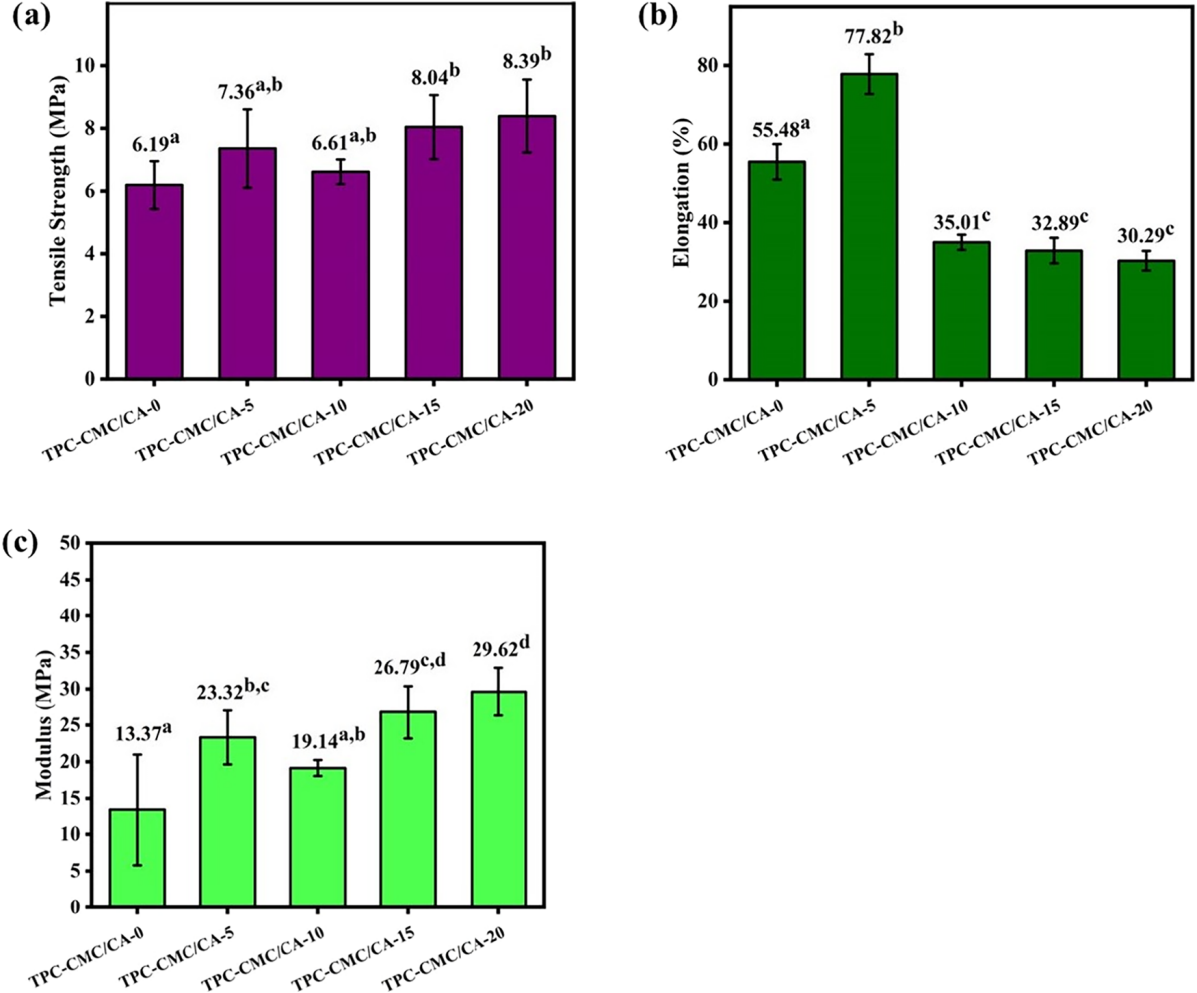
(a) tensile strength, (b) elongation, and (c) modulus for TPC-CMC/CA-0, TPC-CMC/CA-5, TPC-CMC/CA-10, TPC-CMC/CA-15, and TPC-CMC/CA-20. Means followed by equal letters did not differ (*p* < 0.05) by Duncan’s test.

The tensile strength results are shown in Figure 8a, the average values for TPC-CMC/CA-0, TPC-CMC/CA-5, TPC-CMC/CA-10, TPC-CMC/CA-15, and TPC-CMC/CA-20 were 6.19, 7.36, 6.61, 8.04, and 8.39 MPa, respectively. These results indicate that citric acid increased the tensile strength of TPC-CMC films. The TPC-CMC/CA-15 film showed about a 30% increase compared to the film without citric acid. This increase can be attributed to ester bond formation between the OH groups of the polymer matrix and the carboxylic groups of citric acid, making the matrix more rigid and raising σ values.^31^ Our results agree with those for starch films cross-linked with citric acid.^36^ In contrast, films with 80% CMC and 20% cornstarch cross-linked with epichloridine showed decreased tensile strength due to the formation of 3-green propylene glycol, which acts as a plasticizer.^29^

The effect of citric acid on the elongation of TPC-CMC films is shown in Figure 8b. In general, the increase in citric acid content promoted a decrease in the elongation of the films, which is attributed to the presence of citric acid that acts by promoting cross-linking between the polymer chains and thus decreasing their flexibility.^15^ However, the TPC-CMC/CA-5 film demonstrated the highest ε value. At this level, citric acid probably acted as a plasticizer, diminishing intermolecular interactions between starch chains, promoting an increase in ε. Nevertheless, higher acid citric levels may have compromised the structural integrity of the films, resulting in a decrease in ε.^20^ Similar findings have been reported for cornstarch films cross-linked with citric acid.^36^ In contrast to our results, an increase in ε from 9.73 to 59.08% was observed in potato starch/pectin films with 3% bioactive compounds from cashew apples and 0.5% citric acid. The authors attributed this increase to the plasticizing effect of the bioactive compounds, which increased the flexibility of the films.^41^ Thus, our results demonstrate that the addition of citric acid to TPC-CMC films profoundly influences their tensile properties.

Figure 8c shows that the values for the modulus of the films TPC-CMC/CA-0, TPC-CMC/CA-5, TPC-CMC/CA-10, TPC-CMC/CA-15, and TPC-CMC/CA-20 were 13.37, 23.32, 19.14, 26.79, and 29.62 MPa, respectively. A significant increase was observed in all TPC-CMC films with citric acid. This behavior is attributed to cross-linking with citric acid through the formation of ester bonds between the polymer chains, which favors an increase in the modulus.^15^

## 4 Conclusion

TPC-CMC films with different citric acid contents (5, 10, 15, and 20%) were prepared using solvent casting. Citric acid was used as a cross-linking agent. All the films exhibited a high degree of homogeneity with smooth surfaces and no bubbles or cracks. The thickness of the film was approximately 0.10 mm. This behavior was attributed to the strong chemical compatibility between the polymers. The FTIR spectra of the TPC-CMC films with citric acid displayed similar spectral behavior, containing absorption bands typical of starch and CMC. Notably, the films with citric acid showed ester group signals, indicating successful cross-linking between the citric acid carboxyl groups and the hydroxyl groups of the polymer matrix. SEM images revealed a uniform surface morphology and no detectable porosity in the films with citric acid, highlighting the compatibility of the materials. The addition of citric acid to TPC-CMC films decreased water swelling, moisture absorption, and water vapor permeation. This is due to the formation of ester bonds between the carboxyl groups in citric acid and the hydroxyl groups in the matrix. FTIR analysis supported this explanation. The formation of these bonds made the biopolymer matrix more organized and reduced the water susceptibility of the films. All films were highly transparent, which was attributed to their good miscibility. between cornstarch and CMC. Citric acid, as a cross-linking agent, did not affect transparency. These features are well-suited for food packaging, as they allow the visualization of stored food. Furthermore, citric acid increased the tensile strength of TPC-CMC films from 6.07 to 8.39 MPa in TPC-CMC/CA-15. The modulus also increased, rising by approximately 115% in the TPC-CMC/CA-15 film compared to the control film. This improvement was due to cross-linking, which resulted in a more cohesive material with a higher modulus and tensile strength. Our results show that citric acid strongly influences the water susceptibility and tensile properties of TPC-CMC films. Overall, TPC-CMC/CA-15 demonstrated satisfactory performance, indicating its potential for packaging applications

## References

[1] Nair L. S., Laurencin C. T. (2007). Biodegradable Polymers as Biomaterials. Prog. Polym. Sci..

[2] Velasquez S. T. R., Hu Q., Kramm J., Santin V. C., Völker C., Wurm F. R. (2025). Plastics of the
Future? An Interdisciplinary Review
on Biobased and Biodegradable Polymers: Progress in Chemistry, Societal
Views, and Environmental Implications. Angew.
Chem., Int. Ed..

[3] Dallaev R., Papež N., Allaham M. M., Holcman V. (2025). Biodegradable Polymers:
Properties, Applications, and Environmental Impact. Polymers.

[4] Hernández V., Ibarra D., Triana J. F., Martínez-Soto B., Faúndez M., Vasco D. A., Gordillo L., Herrera F., García-Herrera C., Garmulewicz A. (2022). Agar Biopolymer
Films for Biodegradable Packaging: A Reference Dataset for Exploring
the Limits of Mechanical Performance. Materials.

[5] Sancakli A., Basaran B., Arican F., Polat O. (2021). Effects of Bovine Gelatin
Viscosity on Gelatin-Based Edible Film Mechanical, Physical and Morphological
Properties. SN Appl. Sci..

[6] Zheng L., Zhu Z., Pan L., Zhong L., Xiao S., Zhao C., Liu Y., Xu J., Zhang Y. (2025). Polysaccharides and Proteins as Natural
Polymers for Electrospun Wound Dressings: A Review of Healing Potential,
Challenges, and Crosslinking Strategies. Int.
J. Biol. Macromol..

[7] Mali S., Grossmann M. V. E., Yamashita F. (2010). Filmes de amido: produção,
propriedades e potencial de utilização. Semina:Cienc. Agrar..

[8] Wang M., Li W., Zhao F., Deng S., Li T., Wu X., Mu Y., Yang K., Zhang A., Yang X. (2025). A Review on the Application
of Starch in Biodegradable Mulch Films. Int.
J. Biol. Macromol..

[9] Tarique J., Sapuan S. M., Khalina A. (2021). Effect of
Glycerol Plasticizer Loading
on the Physical, Mechanical, Thermal, and Barrier Properties of Arrowroot
(Maranta Arundinacea) Starch Biopolymers. Sci.
Rep..

[10] Md
Nor S., Ding P. (2020). Trends and Advances in Edible Biopolymer Coating for
Tropical Fruit: A Review. Food Res. Int..

[11] Jie X., Lin C., Qian C., He G., Feng Y., Yin X. (2024). Preparation
and Properties of Thermoplastic Starch under the Synergism of Ultrasonic
and Elongational Rheology. Int. J. Biol. Macromol..

[12] Tavares K. M., Campos A. de., Luchesi B. R., Resende A. A., Oliveira J. E. de., Marconcini J. M. (2020). Effect
of Carboxymethyl Cellulose
Concentration on Mechanical and Water Vapor Barrier Properties of
Corn Starch Films. Carbohydr. Polym..

[13] Kurhade R. R., Shaikh M. S., Nagulwar V., Kale M. A. (2025). Advancements in
Carboxymethyl Cellulose (CMC) Modifications and Their Diverse Biomedical
Applications: A Comprehensive Review. Int. J.
Polym. Mater. Polym. Biomater..

[14] Tavares K. M., Campos A. de., Mitsuyuki M. C., Luchesi B. R., Marconcini J. M. (2019). Corn and
Cassava Starch with Carboxymethyl Cellulose Films and Its Mechanical
and Hydrophobic Properties. Carbohydr. Polym..

[15] Nongnual T., Butprom N., Boonsang S., Kaewpirom S. (2024). Citric Acid
Crosslinked Carboxymethyl Cellulose Edible Films: A Case Study on
Preserving Freshness in Bananas. Int. J. Biol.
Macromol..

[16] Kunarbekova M., Shynzhyrbai K., Mataev M., Bexeitova K., Kudaibergenov K., Sailaukhanuly Ye., Azat S., Askaruly K., Tuleshov Ye., Zhantikeyev S. U., Ybyraiymkul D. (2023). Biopolymers
Synthesis and Application. Mater. Today Proc..

[17] Ghorpade V. S., Mali K. K., Dias R. J., Dhawale S. C., Digole R. R., Gandhi J. M., Bobde K. A., Mali R. K. (2024). Citric
Acid Crosslinked
Hydroxyethyl Tamarind Gum-Based Hydrogel Films: A Promising Biomaterial
for Drug Delivery. Int. J. Biol. Macromol..

[18] Wen L., Liang Y., Lin Z., Xie D., Zheng Z., Xu C., Lin B. (2021). Design of Multifunctional
Food Packaging Films Based
on Carboxymethyl Chitosan/Polyvinyl Alcohol Crosslinked Network by
Using Citric Acid as Crosslinker. Polymer.

[19] Thakare N. R., Gogoi P., Bharali P., Hazarika S. (2024). Influence of Copper
Ion Cross-Linked CMC-PVA Film on Cell Viability and Cell Proliferation
Study. Int. J. Biol. Macromol..

[20] Ghanbarzadeh B., Almasi H., Entezami A. A. (2011). Improving
the Barrier and Mechanical
Properties of Corn Starch-Based Edible Films: Effect of Citric Acid
and Carboxymethyl Cellulose. Ind. Crops Prod..

[21] Liu J., Chen J., Dong N., Ming J., Zhao G. (2012). Determination
of Degree of Substitution of Carboxymethyl Starch by Fourier Transform
Mid-Infrared Spectroscopy Coupled with Partial Least Squares. Food Chem..

[22] Standard Test Methods for Water Vapor Transmission of Materials. https://www.astm.org/e0096_e0096m-14.html (accessed Feb 10, 2025).

[23] Peighambardoust S. H., Fasihnia S. H., Peighambardoust S. J., Pateiro M., Domínguez R., Lorenzo J. M. (2021). Active Polypropylene-Based Films Incorporating Combined
Antioxidants and Antimicrobials: Preparation and Characterization. Foods.

[24] Capanema N. S. V., Mansur A. A. P., de Jesus A. C., Carvalho S. M., de Oliveira L. C., Mansur H. S. (2018). Superabsorbent Crosslinked Carboxymethyl Cellulose-PEG
Hydrogels for Potential Wound Dressing Applications. Int. J. Biol. Macromol..

[25] Abdullah A. H. D., Chalimah S., Primadona I., Hanantyo M. H. G. (2018). Physical and
Chemical Properties of Corn, Cassava, and Potato Starchs. IOP Conf. Ser. Earth Environ. Sci..

[26] Pimpang, P. Effect of Concentration of Citric Acid on Size and Optical Properties of Fluorescence Graphene Quantum Dots Prepared by Tuning Carbonization Degree Chiang Mai J. Sci., 45 5 2005 2014.

[27] Priyanka S., Karthick Raja Namasivayam S., Kennedy J. F., Moovendhan M. (2024). Starch-Chitosan-Taro
Mucilage Nanocomposite Active Food Packaging Film Doped with Zinc
Oxide Nanoparticles – Fabrication, Mechanical Properties, Anti-Bacterial
Activity and Eco Toxicity Assessment. Int. J.
Biol. Macromol..

[28] de
Almeida Nascimento J. A., dos Santos A. F., Lima Silva I. D., Lago Falcão E. H., Britto D. de., Vinhas G. M. (2021). Physico-Chemical,
Mechanical and Morphological Properties of Biodegradable Films Based
on Arrowroot Starch and Poly­(Vinyl Alcohol). J. Macromol. Sci. Part B.

[29] Hu G., Lan X., Peng B., Liao J., Xiong Y. (2024). Water Resistant, Biodegradable
and Flexible Corn Starch/Carboxymethyl Cellulose Composite Film for
Slow-Release Fertilizer Coating Materials. Int.
J. Biol. Macromol..

[30] Taharuddin N. H., Jumaidin R., Mansor M. R., Hazrati K. Z., Hafila K. Z., Md Yusof F. A. (2024). Synergistic Effect
of *Hylocereus Polyrhizus* (Dragon Fruit)
Peel on Physicomechanical, Thermal, and Biodegradation
Properties of Thermoplastic Sago Starch/Agar Composites. Int. J. Biol. Macromol..

[31] Yar M. S., Ibeogu I. H., Bako H. K., Alnadari F., Bilal M., Rehman F., Zhu J., Zhou T., Zhao Z., Li C. (2024). A Novel Carboxymethyl
Cellulose/Gum Xanthan and Citric Acid-Based
Film That Enhances the Precision of Blackcurrant Anthocyanin-Induced
Color Detection for Beef Spoilage Tracking. Food Chem..

[32] Skov K. B., Portillo-Perez G. A., Franco M., Martinez M. M. (2025). Impact of Citric
Acid-Mediated Starch Crosslinking on the Properties of Films Derived
from Stale Bread Particles: A Comparative Study of Solvent Casting
and Melt-Mixing/Hot Pressing Processing. Int.
J. Biol. Macromol..

[33] Almeida T., Karamysheva A., Valente B. F. A., Silva J. M., Braz M., Almeida A., Silvestre A. J. D., Vilela C., Freire C. S. R. (2023). Biobased
Ternary Films of Thermoplastic Starch, Bacterial Nanocellulose and
Gallic Acid for Active Food Packaging. Food
Hydrocolloids.

[34] Chandrika K. S. V. P., Singh A., Prasad R. D., Yadav P., Dhara M., Kavya M., Kumar A., Gopalan B. (2025). Porous Crosslinked
CMC-PVA Biopolymer Films: Synthesis, Standardization, and Application
in Seed Coating for Improved Germination. Carbohydr.
Polym. Technol. Appl..

[35] Morais M. A. P., Silva M., Barros M., Halley P., Almeida Y., Vinhas G. (2024). Impact of Citric Acid on Guar Gum
Carboxymethylcellulose
Crosslinked Blend Films. J. Appl. Polym. Sci..

[36] Reddy N., Yang Y. (2010). Citric Acid Cross-Linking
of Starch Films. Food Chem..

[37] Wilpiszewska K., Antosik A. K., Schmidt B., Janik J., Rokicka J. (2020). Hydrophilic
Films Based on Carboxymethylated Derivatives of Starch and Cellulose. Polymers.

[38] Hu W., Zou Z., Li H., Zhang Z., Yu J., Tang Q. (2022). Fabrication
of Highly Transparent and Multifunctional Polyvinyl Alcohol/Starch
Based Nanocomposite Films Using Zinc Oxide Nanoparticles as Compatibilizers. Int. J. Biol. Macromol..

[39] da
Silva Júnior G. S. R., de Vilhena M. B., de Sousa Cunha E. J., da Silva Souza J. A., Del Nero J., Júnior S. A., Mâcedo E. N., Cândido V. S., da Silva C. A. B., da Silva Paula M. V. (2025). Arrowroot Starch/Carboxymethylcellulose-ZnO
NP Films: Thermal, Tensile, and Biodegradation Properties. Polym. Eng. Sci..

[40] Ghorpade V. S., Yadav A. V., Dias R. J., Mali K. K., Pargaonkar S. S., Shinde P. V., Dhane N. S. (2018). Citric
Acid Crosslinked Carboxymethylcellulose-Poly­(Ethylene
Glycol) Hydrogel Films for Delivery of Poorly Soluble Drugs. Int. J. Biol. Macromol..

[41] de
Moura Fernandes J., Grisi C. V. B., da Silva R. D. C. A., da Costa Monção É., de Barros G. A., do Nascimento S. D., Maciel J. F., de Cordeiro A. M. T. M., Queiroz N., Souza A. L. (2025). Antimicrobial Packaging from Potato
Starch and Pectin with Citric Acid and Bioactive Compounds from Cashew
Apple: Preparation, Characterization, and Application in Bread. ACS Omega.

